# Chronic Distress in Male Mice Impairs Motivation Compromising Both Effort and Reward Processing With Altered Anterior Insular Cortex and Basolateral Amygdala Neural Activation

**DOI:** 10.3389/fnbeh.2021.717701

**Published:** 2021-09-13

**Authors:** Lidia Cabeza, Bahrie Ramadan, Stephanie Cramoisy, Christophe Houdayer, Emmanuel Haffen, Pierre-Yves Risold, Dominique Fellmann, Yvan Peterschmitt

**Affiliations:** ^1^Laboratoire de Recherches Intégratives en Neurosciences et Psychologie Cognitive, Université de Bourgogne – Franche-Comté, Besançon, France; ^2^Clinical Psychiatry, Hôpital Universitaire CHRU, Besançon, France; ^3^CIC-INSERM-1431, Hôpital Universitaire CHRU, Besançon, France

**Keywords:** chronic distress, motivation, reward processing, effort, glucocorticoids, insular cortex, basolateral amygdala

## Abstract

In humans and mammals, effort-based decision-making for monetary or food rewards paradigms contributes to the study of adaptive goal-directed behaviours acquired through reinforcement learning. Chronic distress modelled by repeated exposure to glucocorticoids in rodents induces suboptimal decision-making under uncertainty by impinging on instrumental acquisition and prompting negative valence behaviours. In order to further disentangle the motivational tenets of adaptive decision-making, this study addressed the consequences of enduring distress on relevant effort and reward-processing dimensions. Experimentally, appetitive and consummatory components of motivation were evaluated in adult C57BL/6JRj male mice experiencing chronic distress induced by oral corticosterone (CORT), using multiple complementary discrete behavioural tests. Behavioural data (from novelty suppressed feeding, operant effort-based choice, free feeding, and sucrose preference tasks) collectively show that behavioural initiation, effort allocation, and hedonic appreciation and valuation are altered in mice exposed to several weeks of oral CORT treatment. Additionally, data analysis from FosB immunohistochemical processing of postmortem brain samples highlights CORT-dependent dampening of neural activation in the anterior insular cortex (aIC) and basolateral amygdala (BLA), key telencephalic brain regions involved in appetitive and consummatory motivational processing. Combined, these results suggest that chronic distress-induced irregular aIC and BLA neural activations with reduced effort production and attenuated reward value processing during reinforcement-based instrumental learning could result in maladaptive decision-making under uncertainty. The current study further illustrates how effort and reward processing contribute to adjust the motivational threshold triggering goal-directed behaviours in versatile environments.

## Introduction

Motivation is defined as “a reason or reasons for acting or behaving in a particular way” (Husain and Roiser, 2018), and is often understood as a measure of the amount of energy or other resources that an individual is willing to invest in achieving a valued outcome (Berridge, 2004). Motivation is, therefore, considered a critical modulator of reward-related behaviour, which may impact high-order functioning, including decision-making. Motivational state of individuals, therefore, modulates their engagement in rewarding behaviours, from initiation (*appetitive behaviour*) to cessation with maintenance through persistent effort (Husain and Roiser, 2018; Salamone et al., 2018).

Apathy or loss of motivation in goal-directed behaviour, attributable to emotional disturbance (Marin, 1990, 1991), has been described as a core symptom across psychiatric disorders subsequent to chronic distress (Pedersen et al., 2009; Hartmann et al., 2015; Husain and Roiser, 2018), including depression (Treadway and Zald, 2011; Treadway et al., 2012). Depressed patients, even if able to experience pleasure *per se*, are usually reluctant to expend effort in exchange for rewarding experiences (Marin, 1990). In combination with energy-related deficiencies or *anergia*, or even with fatigue, overestimation of the effort necessary for obtaining rewards and reduced reward anticipation could be at the origin of motivational deficits in this clinical population, potentially leading to a suboptimal integration of cost/benefit information (Treadway and Zald, 2011). Consequently, disruptions in reward processing influence the establishment of adaptive action-outcome associations that lead to the development of interest, desire, and anticipation for the latter (Kring and Barch, 2014; Rizvi et al., 2016). Impaired associative learning reported in psychiatric conditions (Horan et al., 2006; Der-Avakian and Markou, 2012; Griffiths et al., 2014) might result from *abulia* (i.e., reduced ability to initiate an action) or *anhedonia* (i.e., reduced ability to feel pleasure or lack of reactivity to positive experience from reward), but they are likely not the only contributors (Marin, 1990; Pizzagalli, 2014; Husain and Roiser, 2018).Therefore, reward anticipation, which involves momentary motivational arousal in healthy conditions, effort valuation to obtain a reward, and motivation in a broad picture are, among others, processes involved in hedonic appreciation that may lead to variability in goal-directed behavioural outputs.

Behavioural adjustment to novel situations is necessary in order to adaptively modify behaviour in dynamic environments and requires information updating and monitoring necessary for effective cue processing and strategy definition (Seu et al., 2009; Dajani and Uddin, 2015; Uddin, 2021). Consequently, deficits in these processes considerably impact the ability of individuals to learn from the outcomes of their actions and might decrease the quality of their decisions.

Preclinical studies on the effect of chronic distress have evidenced motivational deficits *via* appetitive and effort-related tasks, and, in particular, chronic glucocorticoids (GC) administration has been demonstrated to interfere in the instrumental conditioned responding of rodents to food reward (Gourley et al., 2008; Olausson et al., 2013; Dieterich et al., 2019, 2020). However, these alterations seem to be highly dependent upon type, intensity, and duration of the stressors (Hurtubise and Howland, 2017). Chronic GC administration in mice efficiently induces negative valence behaviours reminiscent to those subsequent to human chronic distress (Dieterich et al., 2019; Cabeza et al., 2021). Yet, results are controversial concerning the impairment of positive valence behaviours (David et al., 2009; Olausson et al., 2013; Dieterich et al., 2019; Cabeza et al., 2021). However, recent results from our laboratory have demonstrated that chronic exposure to GC disrupts male mice decision-making in a valued-based gambling task, mostly by obstructing the exploration-exploitation trade-off.

Several brain regions and networks are suggested to be involved in decisional processing under distress. The present study, therefore, aims at disentangling the neural underpinnings of the relationship between chronic GC exposure and motivational deficits in order to obtain insight into the behavioural and neural mechanisms underlying suboptimal decision-making upon distress. For that, the neural activation pattern of multiple discrete brain areas has been explored in relation to appetitive and consummatory motivational behaviours. We forecasted aberrant neural activation patterns in mice after chronic GC administration, especially in (i) the medial prefrontal cortex (mPFC, prelimbic–PL, and infralimbic–IL, cortices), given its role in instrumental acquisition and appetitive behaviours, for its contribution to reward anticipation and processing, and for its participation in the stress response (Delatour and Gisquet-Verrier, 2000; Akana et al., 2001; Capuzzo and Floresco, 2020); (ii) we also expected the orbitofrontal cortex (OFC) to play a role since it directly participates in effort modulation, and also for its contribution to subjective outcome valuation and contextual salient information integration (Stalnaker et al., 2009; Gourley et al., 2010); (iii) we prognosticated aberrant neural activity within the nucleus accumbens (NAc) – basolateral amygdala (BLA) – ventral tegmental area (VTA) network, which participates in multiple aspects of motivational processing, including reward processing and integration of incentive value dynamics (Wang S.H., 2005; Roozendaal et al., 2009; Floresco, 2015; Wassum and Izquierdo, 2015; Piantadosi et al., 2017; Bouarab et al., 2019); and (iv) we expected to evidence a significant contribution of the anterior insular cortex (aIC), given its role in interoceptive information and effort-based processing, and for its contribution to the representation of anticipatory cues and decision-making (Nieuwenhuys, 2012; Daniel et al., 2017; Gogolla, 2017).

Since the emergence of several neuropsychiatric disorders has been related to the deleterious effects of sustained distress, understanding the underlying mechanisms of the co-occurring motivational deficits may help identify predictive biomarkers for treatment selection toward precision medicine in biological psychiatry.

## Materials and Methods

### Animals

**FIGURE 1:**
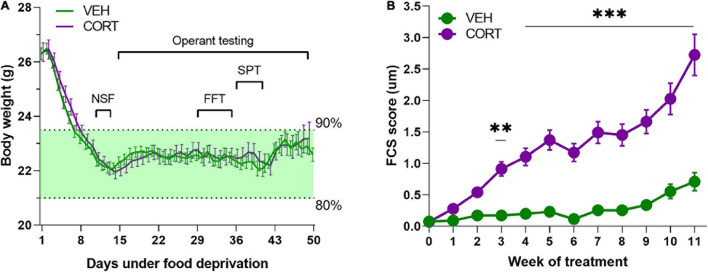
**(A)** Body weight monitoring of animals during sustained food deprivation. Mice required approximately a week of food deprivation to reach the weight-limit criteria (90–80% of free feeding weight). Animals from both pharmacological conditions (VEH, *n* = 61; CORT, *n* = 61) did not significantly differ in their body weight irrespective of the experimental time point [*F*_2,120_ = 0.001, *p* = 0.97]. **(B)** Evolution of the FCS of animals chronically treated with VEH and CORT. Corticosterone administration in mice (*n* = 61) provokes significant FCS degradation from the 3rd week of treatment (**p < 0.01; ***p < 0.001), compared with the control group (VEH, *n* = 61).

One hundred and twenty-two 6- to 8-week-old male C57BL/6JRj mice (*EtsJanvier Labs*, Saint-Berthevin, France) were group housed and maintained under a normal 12-h light/dark cycle with constant temperature (22 ± 2°C). They had access to standard food (KlibaNafag3430PMS10, *Serlab*, CH-4303 Kaiserau, Germany) *ad libitum* up to at least 1 week before the start of the behavioural evaluations, and from then onward, they were under food deprivation from 80 to 90% of their free-feeding weight [mean ± SEM (g) = 26.33 ± 0.20] (see Figure 1A for details). Bottles containing water and/or treatment were available at all times.

The experiments were all conducted following the standards of the Ethical Committee in Animal Experimentation from Besançon (CEBEA-58; A-25-056-2). All efforts were made to minimise animal suffering during the experiments according to the Directive from the European Council at the 22nd of September 2010 (2010/63/EU).

### Pharmacological Treatment

Behavioural assessments started from the 6th week of treatment. Half the individuals received corticosterone (CORT,-4-Pregnene-11β-diol-3,20-dione-21-dione, *Sigma-Aldrich*, France) dissolved in a vehicle (VEH, 0.45% hydroxypropyl-β-cyclodextrin-βCD, *Roquette GmbH*, France) in the drinking water (35 μg/ml equivalent to 5 mg/kg/day, CORT group, *n* = 61) throughout the entire experiment. The other half of the animals received vehicle in the drinking water (VEH group). Bottles containing CORT or VEH were prepared twice a week (David et al., 2009; Mekiri et al., 2017).

### Behavioural Characterisation

Behavioural evaluations were conducted during the light phase of the cycle (from 8:00 a.m.). The animals were randomly separated in two groups (A and B) in order to evaluate different motivational components at similar times of treatment. Group A comprised 42 animals (VEH, *n* = 21; CORT, *n* = 21), which were first tested in the novelty suppressed feeding (NSF) task, and then in an operant progressive ratio (PR) schedule of reinforcement task. The animals from group B (VEH, *n* = 40; CORT, *n* = 40) were tested in the free feeding task (FFT) and the sucrose preference test (SPT). Different foods were used as rewards: standard chow was used in the NSF task; 20-mg grain-based pellets (Dustless Precision Pellets^®^ Grain-Based Diet, *PHYMEP s.a.r.L.*, Paris, France) were used in the PR task and the FFT; ∼20-mg chocolate pellets (Choco Pops, *Kellogg’s*^®^) were also presented during the FFT; 2.5 and 0.8% sucrose solutions [D(+)Saccharose, *Carl Roth*, Karlsruhe, Germany] were used for the SPT.

#### Fur Coat State Evaluation

The state of the fur coat frequently degrades in rodents subjected to sustained distress. Its aspect is frequently used as an index of the quality of self-oriented behaviour and practice of cleaning of animals (Nollet et al., 2013), but scientific evidence has also reported adverse effects of chronic exposure to exogenous GCs on epidermal homeostasis (Pérez, 2011) and hair growth initiation (Stenn et al., 1993).

As an index of the efficacy of the treatment and the quality of self-oriented behaviour, the coat state of the animals was evaluated weekly from the beginning of the experiment. For this, seven body regions were rated (head, neck, abdomen, front legs, hind legs, and tail), and a score between 0 and 1 was assigned to each one as described by Nollet et al., 2013 (0: good state; 0.5: coat moderately degraded; 1: bad state). A final score between 0 and 7 was calculated, adding all scores.

#### Novelty Suppressed Feeding Task

The novelty suppressed feeding (NSF) task evaluates hyponeophagia in rodents (i.e., the inhibition of feeding produced by a novel environment) and has been proved useful to evaluate the emergence of negative valence behaviour in rodents (Samuels and Hen, 2011). During the 6th week of differential treatment, the food-deprived animals were individually placed in an opaque cylindrical open-field apparatus (diameter: 47 cm; height: 60 cm), in the centre of which two grain-based pellets were located on a white piece of filter paper (diameter: 10 cm). The latency of the animals to start eating was recorded and used as an index of appetitive behaviour. The test finished immediately after the animals started to eat or when a maximum time of 15 min elapsed. Immediately after the test, the animals were transferred to an individual home cage where food was available, and the total amount of food consumed during 5 min was measured.

#### Operant Habituation and Training

Operant behaviour was evaluated from the 7th week of differential treatment, using five identical two-hole-operant chambers (*Med Associates*, Hertfordshire, United Kingdom), individually housed inside sound-attenuating cubicles with ventilating fans and connected to a PC computer with MedPC-5 software *via* a *Smart Control* interface cabinet. The MedPC programmes used in this study were written in Medstate notation code.

Prior to behavioural evaluation, the animals followed a habituation period and operant training. *Habituation*: the animals were introduced during 10 min for three consecutive days in the operant chambers, where they could explore an empty and illuminated food receptacle in one side, and two unilluminated identical holes in the opposite side. On the 3rd day, they could find food in the food receptacle. *Training*: following the habituation sessions, the mice were trained to nose-poke in one of the holes on a fixed ratio (FR)-1 schedule of reinforcement, where a single nose-poke elicited the delivery of a food pellet. Only the lit hole was designated as “active” and triggered the reward delivery. The allocation of the active and inactive holes was random for each mouse, except for the animals displaying spatial preference during the habituation period (two times as frequent visits to a hole respective to the other). For those, the active hole was selected against their preference. Five-s timeout to the FR-1 was given after a nose-poke, for both, right or wrong choices. During the timeout, additional nose-pokes did not result in reward delivery, giving the mice the time to consume the food. Each training session lasted an hour, or until a maximum of 50 rewards was delivered. The animals were considered to acquire a food-maintained operant responding (*acquisition criteria*) when they (1) exhibited a discrimination index of ≥3:1 for the active versus inactive responses, (2) obtaining ≥20 rewards per session (3) over three consecutive sessions. When these acquisition criteria were fulfilled, the schedule was increased to FR-5, for which five active nose-pokes triggered the delivery of one food reinforcer. The FR-5 training lasted three consecutive days.

#### Progressive Ratio Schedule of Reinforcement

Effort allocation was evaluated using an operant PR protocol (adapted from Sharma et al., 2012) that started after completion of the operant training. The ratio schedule for the PR testing was calculated as previously described (Richardson and Roberts, 1996) using the following formula: [5e^(^*^*R*^*^ × 0.2)^]− 5, where *R* is the number of rewards already earned plus 1. The ratios to obtain a food reward are: 1, 2, 4, 6, 9, 12, 15, 20, 25, 32, 40, 50, 62, 77, 95, and so on. The last ratio completed by an animal is its breakpoint (BP). A PR session lasted an hour or ended after 10 min of inactivity (absence of visits to the holes or the food receptacle). The PR testing finished when the number of rewards earned in a session deviated ≤10% for three consecutive days.

#### Free Feeding Task

This behavioural paradigm (modified from Vichaya et al., 2014) allows investigating the contributions of positive valence systems to decision-making by focusing on hedonic appreciation and valuation. From the 8th week of differential treatment, the animals were free to access and feed from two different sorts of nourishment, including a sweet option appealing to the natural preference of the animals for sweetness. Subjective valuation of the presented options is addressed by the choice itself (i.e., food reward primarily chosen for consumption), while appetitive behaviour is evaluated from the latency until the animals are starting to eat. Subsequently, hedonic appreciation is approached through consummatory behaviour of the animals. Cognitive aspects contributing to behavioural initiation, such as behavioural flexibility or risk-taking, are inferred from a variation of the paradigm for food neophobia evaluation (i.e., presentation of one unfamiliar sort of food).

The mice were placed in a transparent cage where two receptacles with food pellets were exposed, one with 10 grain-based pellets and the other one, with 10 chocolate pellets. The nature of the first pellet chosen, as well as the latency of this first choice, and the total number of pellets consumed were noted. The test finished when all pieces of the first choice were eaten, or after maximal test duration of 5 min. The animals that failed to choose within the assigned time were considered as *undecided*. The mice were tested twice in the FFT, 1 week apart: at the beginning of the neophobia evaluation, the animals were familiar with the grain-based pellets but not with the chocolate option, while during the second evaluation (referred as “re-test”), all the animals were familiar with both types of rewards.

#### Sucrose Preference Test

Hedonic appreciation was addressed by studying the preference of 2.5 and 0.8% sucrose solutions over water of animals VEH/CORT treated during 10 weeks. Founded upon the natural preference of animals for sweetness (Berridge, 1996, 2004), this paradigm allows investigating hedonic aspects of motivated behaviours, also referred as “liking phase” (Husain and Roiser, 2018) through consummatory actions.

The sucrose preference test (SPT) lasted four consecutive nights (one night of forced sucrose consumption and three nights of free choice) and was performed as previously described (Cabeza et al., 2021). The sucrose preference was calculated as the percentage of sucrose solution consumption relative to the total liquid intake during the last session. Two different sucrose concentrations were used in order to prevent potential ceiling effects.

### Animals Sacrifice and Brain Sampling

Twelve mice (VEH, *n* = 6; CORT, *n* = 6) randomly chosen from the behavioural group A were sacrificed 24 h following the attainment of their BP in the PR testing for FosB immunostaining. The animals were deeply anesthetised with an intraperitoneal injection of Dolethal (1 ml/kg, *Vetoquinol*), then transcardially perfused with 0.9% NaCl, followed by ice-cold 4% paraformaldehyde (PFA, *Roth*^®^, Karlsruhe, Germany), fixative in 0.1 M phosphate buffer pH 7.4. Once extracted, brains were postfixed overnight in the same fixative at 4°C and cryoprotected by immersion in a 15% sucrose solution (D(+)-Saccharose, *Roth*^®^, Karlsruhe, Germany) in a 0.1 M phosphate buffer 24 h at 4°C. The brains were then frozen by immersion in isopentane (2-methylbutane, *Roth*^®^, Karlsruhe, Germany) at −74°C using a Snap-Frost^TM^ system (*Excilone*, France) and stored at −80°C until processed. The brains were cut in coronal 30-μm-thick serial sections, collected in a cryoprotector solution (1:1:2 glycerol/ethylene glycol/phosphate buffered saline – PBS; *Roth*^®^, Karlsruhe, Germany) and stored at −40°C.

### Immunohistochemistry

Several sections were selected in order to study the following brain structures: mPFC (PL and IL areas), OFC and NAc (core – NAcC, and shell -NAcS) at (1.94–1.70) anterior to Bregma (aB); rostral aIC at (0.86–0.50) and caudal at (0.26–0.02) aB; paraventricular nucleus of the hypothalamus (PVN) and dorsal region of the bed nucleus of the stria terminalis (BNST) at (0.26–0.02) aB; amygdala (central -CeA and BLA) at (0.94–1.34) posterior to Bregma (pB); parasubthalamic nucleus (PSTN) at (2.06–2.30) pB; and VTA at (2.92–3.16) pB (according to Franklin and Paxinos, 2008). Once mounted on gelatin-coated slides, the sections were exposed to an antigen retrieval method to maximise antigenic site exposure for antibody binding. The sections were immersed for 40 min in 10-mM citrate buffer pH 6 (sodium citrate, *Sigma Aldrich*, Germany), previously heated at 96°C in a water bath. Then, the buffer was left to cool down at room temperature before removing the sections. After several repeats of washing, the sections were exposed 15 min to a 0.3% hydrogen peroxide solution in order to block endogenous peroxidase activity, avoiding nonspecific background staining. Next, the sections were incubated with the primary antibody (*Sc-48*, rabbit anti-FosB, *Santa Cruz Biotechnology*, 1:500) during 48 h at room temperature. Tissues were then incubated with the secondary HRP antibody (*BA-1,000*, goat anti-rabbit Ig, *Vector Laboratories*, 1:1,500) during 24 h at room temperature. After washing, the amplification of the signal was addressed by using an avidin horseradish peroxidase complex (ABC Elite kit, *Vector Laboratories*) for 40 min at room temperature. A 3,3′-Diaminobenzidine (DAB) chromogen solution was used to visualise the peroxidase complex. The brain sections were then dehydrated with successive alcohol baths (70°, 95°, 100°, and 100°, 3 min), cleared with xylene (*Avantor*^®^, 3 × 5 min) and finally coverslipped with Canada Balsam (*Roth*^®^, Karlsruhe, Germany). Microscopic brain images from enzymatic immunohistochemistry were acquired using 4 × objectives of an Olympus microscope B × 51, equipped with a camera Olympus DP50. *ImageJ* software was used to count FosB-labelled cell nuclei over the regions of interest. Due to technical issues, some sections and related regions could not be included in the final analysis, so that final samples sizes were: PL *n* = 12, IL *n* = 12, OFC *n* = 12, NAc *n* = 12, rostral aIC *n* = 12, caudal aIC *n* = 11, dorsal BNST *n* = 12, PVN *n* = 11, amygdala *n* = 12, PSTN *n* = 10, and VTA *n* = 11.

### Data and Statistical Analyses

Data are presented as means ± SEM.

Statistical analyses were conducted using STATISTICA 10 (*Statsoft*, Palo Alto, United States), and figures were designed using GraphPad Prism 9 software (*GraphPad Inc.*, San Diego, United States). The sample sizes were identified *a priori* by statistical power analysis (G*Power software, Heinrich Heine Universität, Dusseldorf, Germany). Our behavioural sample is predicted to yield highly reproducible outcomes with 1 – β > 0.8 and α < 0.05. Assumptions for parametric analysis were verified prior to each analysis: normality of distribution with Shapiro-Wilk, homogeneity of variance with Leven’s and sphericity with Mauchly’s tests. Behavioural and immunohistological scores from the pharmacological conditions were compared by Student *t*-tests, and when datasets did not meet assumptions for parametric analyses, Mann-Whitney *U* and Wilcoxon tests were used. Behavioural time-dependent measures assessed during the operant testing were analysed by repeated measures ANOVA (RM-ANOVA) with sessions (1–3) as a within-subject factor and treatment (VEH and CORT) as between-subject factors.

Survival curves for NSF scores were compared using the Gehan-Breslow-Wilcoxon (GBW-*X*^2^) method. The assumption of independent and normal distribution of treatment populations within FFT clusters based on differential choice was tested with Chi-squared tests (*X*^2^). Dimensional relationships between various behavioural scores and between behavioural scores and FosB expression in the various brain structures under investigation were analysed using Pearson correlations. For all analyses, the significance level was *p* < 0.05, and analysis with *p* ≤ 0.1 was described as trends. Effect sizes are reported as partial η^2^ (pη^2^).

## Results

Motivational dimensions potentially impacting decision-making under distress were addressed through various behavioural evaluations. The groups of animals, which were under sustained food deprivation, did not significantly differ in their body weight during the complete duration of the experimental assessment (treatment: *F*_2,120_ = 0.001, *p* = 0.97), as it is illustrated in Figure 1A. The weekly evaluation of the FCS of the animals served information about the pharmacological efficacy (pharmacodynamics) and also their self-oriented behaviour (Nollet et al., 2013). Along with the literature, the FCS of CORT-treated mice (*n* = 61) appeared significantly degraded from the 3rd week of treatment compared with the control group (*n* = 61) (main effect of treatment: *F*_1,1279_ = 454.8, *p* < 0.00, pη^2^ = 0.26; week: *F*_13,1279_ = 25, *p* < 0.00, pη^2^ = 0.20; treatment × week interaction: *F*_13,1279_ = 14.3, *p* < 0.00, pη^2^ = 0.13; from the 3rd week of treatment: all *ps* < 0.01, *post hoc*; Figure 1B).

### Group A

**FIGURE 2:**
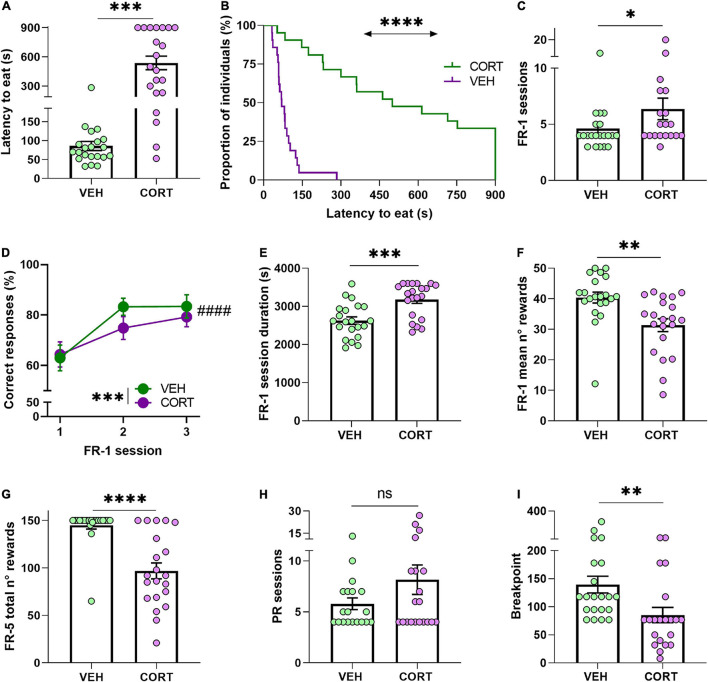
Chronic CORT administration hampers appetitive behaviour in mice. **(A,B)** In the NSF test and under a sustained food restriction protocol, the animals chronically treated with CORT took longer to start eating from a food pellet placed in the centre of an open arena (***p < 0.001; ****p < 0.0001). **(C)** Compared with the control animals, CORT-treated mice required more sessions to reach the acquisition criteria in the FR-1 reinforcement phase, indicating a detrimental effect of the GC pharmacological treatment in action-outcome association (*p < 0.05). **(D)** Animals of both conditions improved the proportion of correct responses during the three first FR-1 sessions (^####^*p* = 0.0000), but the overall performance of the CORT-treated animals was less accurate than in the control animals (***p < 0.001). **(E,F)** Compared with the control mice, the CORT-treated mice required significantly more time to complete the training FR-1 sessions (***p < 0.001), and **(G)** earned significantly less rewards (**p < 0.01). During the transitional FR-5 phase, the CORT-treated animals also obtained significantly less food rewards than the control animals (****p = 0.0000). **(H)** All animals, irrespective of their condition, required a similar number of sessions to reach their BP in the PR testing (ns, not significant). **(I)** However, the CORT-treated animals significantly allocated less effort to obtain food reward, indicating a deleterious effect of the GC pharmacological treatment on reward and effort valuation and processing (**p < 0.01).

The NSF task was used to study the appetitive dimension of motivation by approaching behavioural initiation to feed. Mice treated with CORT during 6 weeks approached and started eating from the presented food after a significantly longer latency period [*n* = 21; mean latency to eat (s) ± SEM: 537.27 ± 69.74] than VEH-treated animals (*n* = 21; 85.63 ± 11.89) (UMW: *Z* = −4.8, *p* = 0.0000; GBW, *X*^2^ = 28, *p* < 0.0001). CORT-treated mice, therefore, displayed a typical negative valence behaviour in the NSF task in accord with the literature (David et al., 2009; Dieterich et al., 2019). Of note, the comparison of the food intake after the test, frequently used as appetite control between groups (David et al., 2009), shows an effect of the treatment with CORT-treated animals eating significantly less [mean food intake (g) ± SEM:0.09 ± 0.02] than VEH animals (0.04 ± 0.02) (*Z* = 3.9, *p* < 0.0001). Since the body weight was monitored for all the animals throughout the food deprivation procedure, this difference can be attributed to CORT-induced metabolic effects preferentially (Wang M., 2005) rather than to differential hunger pressure. See Figures 2A,B.

The contingent association of a nose-poke/action – outcome/reward delivery (i.e., instrumental acquisition) was then evaluated from FR-1 data. The comparison revealed that chronic CORT administration resulted in the animals requiring more sessions to comply with the acquisition criteria [mean of total number of FR-1 sessions ± SEM, VEH: 4.62 ± 0.51; CORT: 6.37 ± 0.96] (UMW: *Z* = −2, *p* < 0.05; Figure 2C), in agreement with previous studies (see, for instance, Dieterich et al., 2019). To complement this result, the percentage of correct responses made by the animals across the three first FR-1 sessions was also compared across the different conditions. A RM-ANOVA revealed a learning effect across the sessions (main effect of session: *F*_2,80_ = 65.7, *p* = 0.0000, pη^2^ = 0.62) and a significant reduced number of correct responses resulting from the pharmacological treatment [mean number of correct responses ± SEM, VEH: 110.33 ± 7.42; CORT: 72.24 ± 7.23] (treatment: *F*_1,40_ = 13.5, *p* < 0.001, pη^2^ = 0.25). Contrary to expectations, a significant interaction between factors was not revealed (session × treatment: *F*_2,80_ = 0.69, *p* = 0.50, pη^2^ = 0.02; Figure 2D). The mean duration of the daily FR-1 sessions was also compared between conditions, showing a significant effect of the treatment on the time that the animals required to complete them [mean duration of FR-1 sessions (s) ± SEM, VEH: 2,629.95 ± 97.97; CORT: 3,178.98 ± 98.71] (*Z* = −3.3, *p* < 0.001; Figure 2E). Similarly, the mean number of rewards earned in the FR-1 training sessions was significantly lower in CORT-treated animals [31.36 ± 2.09] compared with control animals (40.41 ± 1.78) (*Z* = 3.2, *p* < 0.01; Figure 2F). Combined, these results suggest that chronic CORT exposure delays the formation of action-outcome contingencies in operant tasks.

Preceding the PR testing to study effort allocation, mice behaviour was evaluated in a transitional FR-5 schedule of reinforcement. The quantity of obtained rewards was found to be influenced by the GC pharmacological treatment (main effect of treatment: *F*_1,40_ = 26.3, *p* = 0.0000, pη^2^ = 0.40), with CORT-treated animals [mean number of rewards ± SEM: 96.86 ± 8.45], obtaining a smaller amount of food than controls animals [144.95 ± 4.06] (Figure 2G), but without significant effect of the session (session: *F*_2,80_ = 1.73, *p* = 0.18, pη^2^ = 0.04). CORT- and VEH-treated animals also differed in the percentage of correct responses across the FR-5 reinforcement phase (treatment: *F*_1,40_ = 9.7, *p* < 0.01, pη^2^ = 0.19), although both groups responded almost always correctly [mean percentage of correct responses ± SEM, VEH: 93.84 ± 0.98; CORT: 89.67 ± 0.92]. However, these differences need careful interpretation and are not to be directly attributed to the motivational state of the animals, since intrasession satiation might have influenced the behaviour of animals.

The explicit study of motivation to expend effort for a food reinforcer during the PR testing revealed a significant deleterious effect of the pharmacological treatment, again in line with previous studies (Olausson et al., 2013; Dieterich et al., 2019). The animals were tested daily on the PR schedule of reinforcement until they stabilised their performances, i.e., they reached their BP, and no difference between pharmacological conditions was found for the number of required sessions [mean number of sessions ± SEM, VEH: 5.79 ± 0.57; CORT: 8.15 ± 1.45] (UMW, *Z* = −0.6, *p* = 0.57; Figure 2H). Congruent with the scientific literature, these results showed that mice chronically treated with CORT had a lower BP in the PR task [mean BP ± SEM, CORT: 85.33 ± 13.62] compared with the animals from the control group [VEH: 139.57 ± 14.96] (UMW: Z = 3.2, *p* < 0.01) (Figure 2I). In other words, chronic CORT administration lessened the motivation of the animals to allocate effort for food rewards, in agreement with previous findings on altered reward and effort valuation (see, for instance, Gourley et al., 2008).

**FIGURE 3:**
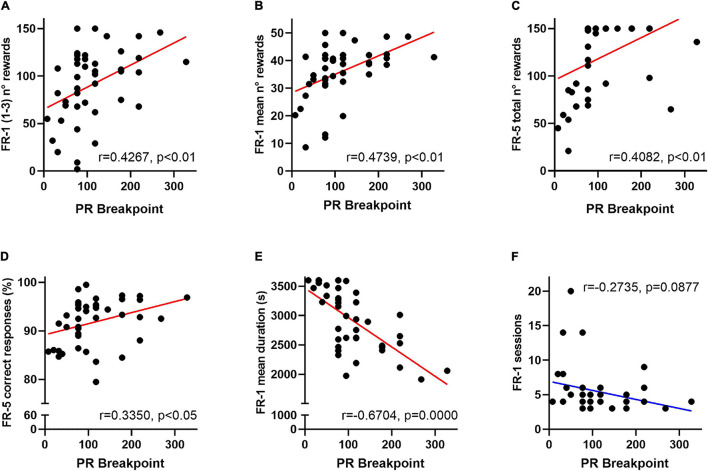
Behavioural FR-1 and FR-5 operant scores predict motivation for effort allocation in mice. Among overall data (VEH- and CORT-treated mice), breakpoints significantly correlate with **(A)** the amount of earned rewards during the three first FR-1 sessions (*p* < 0.01); **(B)** the mean number of rewards earned per FR-1 session (*p* < 0.01) and **(C)** the total amount of earned rewards during the FR-5 (*p* < 0.01). Breakpoint scores also significantly correlate with **(D)** the mean percentage of correct responses (*p* < 0.05) during the FR-5 phase and negatively correlate with **(E)** the mean duration of FR-1 sessions (*p* = 0.0000). **(F)** A negative correlation trend was also identified between breakpoint scores and the number of FR-1 sessions needed to reach instrumental acquisition (*p* = 0.09). Together, these results point to the predictive value of operant training scores for the motivational dimension of effort allocation in the PR schedule of a reinforcement task.

In order to evaluate the putative effect of a delayed instrumental acquisition on effort allocation, FR-1 and FR-5 behavioural scores were subsequently compared in a correlational study. Breakpoints of VEH- and CORT-treated animals significantly correlate with the amount of rewards earned during the first three FR-1 sessions (*r* = 0.4267, *p* < 0.01), the mean number of rewards earned per FR-1 session (*r* = 0.4739, *p* < 0.01), and the total amount of earned rewards (*r* = 0.4082, *p* < 0.01) and the mean percentage of correct responses (*r* = 0.3350, *p* < 0.05) during the FR-5 phase. Breakpoints also negatively correlate with the mean duration of FR-1 sessions (*r* = −0.6704, *p* = 0.0000) and tend to negatively correlate with the number of FR-1 sessions needed to reach instrumental acquisition (*r* = −0.2735, *p* = 0.09). Together, these results suggest that deficits in the motivational dimension of effort allocation could be originated or exacerbated by impaired formation of action-outcome association during training phases. See Figures 3A–F.

### Group B

**FIGURE 4:**
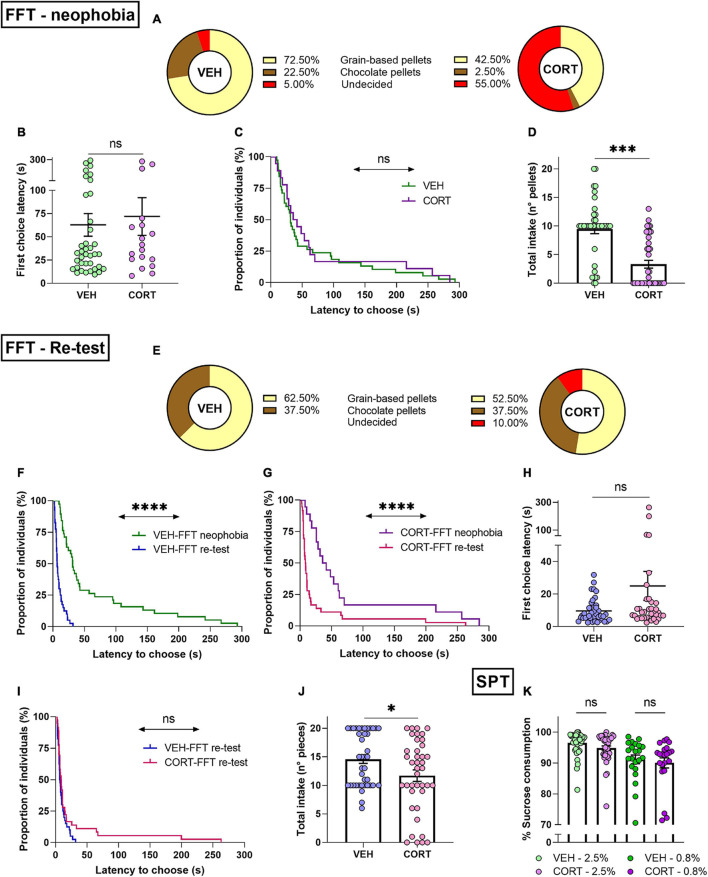
Chronic CORT administration impacts behavioural initiation and consummatory behaviour in mice. **(A)** Food neophobia is emphasised after CORT exposure in the FFT. Different frequency distributions of VEH- and CORT-treated individuals in their choices between familiar (grain-based pellets, VEH: *n* = 29; CORT: *n* = 17) and unfamiliar food rewards (chocolate pellets, VEH: *n* = 9; CORT: *n* = 1). Chronic CORT administration affects appetitive behaviour through an increment of the proportion of animals failing to choose between rewards within the test duration (undecided, VEH: *n* = 2; CORT, *n* = 22). **(B)** During the neophobia approach of the FFT, the VEH-, and CORT-treated animals needed the same amount of time to make a choice and to start eating. **(C)** Appetitive behaviour of animals that eat (independently of their choices) was not affected by the CORT treatment, as shown by the negligible difference in proportions of individuals starting to eat. **(D)** The CORT-treated mice ate significantly less than the control animals during the FFT. **(E)** Chronic CORT exposure does not affect reward valuation but impinges on appetitive behaviour in the FFT re-testing. Different frequency distributions of VEH- and CORT-treated individuals in their choices between grain-based pellets (VEH: *n* = 25; CORT: *n* = 21) and chocolate pellets (VEH: *n* = 15; CORT: *n* = 15). Chronic CORT administration affects appetitive behaviour, as evidenced through an increment of the undecided class (undecided, VEH: *n* = 0; CORT, *n* = 4). **(F)** Animals from the control group started eating significantly earlier in the FFT-re-testing **(G)** as well as the CORT-treated animals, indicating neophobia overcoming. **(H)** In the FFT re-testing, VEH- and CORT-treated animals did not differ in their latency to choose between the two options and start eating (data from undecided individuals excluded). **(I)** The VEH- and CORT-treated individuals displayed similar appetitive behaviour (independently of their choices), as shown by the lack of difference in the proportion of the individuals starting to eat in the FFT re-testing. **(J)** Effect of the CORT treatment on mice consummatory behaviour, with the treated animals eating significantly less than the control animals. **(K)** Chronic CORT administration does not significantly influence hedonic appreciation through consummatory behaviour in the SPT. Mice chronically treated with CORT showed a significant preference for the sweet solution compared with water [2.5%, *n* = 40% of sucrose vs 50% solution: *t*_39_ = 61.6, *p* < 0.00], as the control animals [2.5%, VEH, *n* = 40: *t*_39_ = 79.3, *p* < 0.00], and irrespective of the sucrose concentration [.8%, CORT, *n* = 22: *Z* = 4.1 *p* < 0.0001; VEH, *n* = 22: *Z* = 4.1, *p* < 0.0001] (ns, not significant; *p < 0.05; ***p < 0.001; ****p < 0.0001).

The FFT paradigm used to assess reward appreciation allowed to simultaneously address the influence of food neophobia in mice (VEH, *n* = 40; CORT, *n* = 40). The results show that the majority of individuals chose preferably the familiar grain-based option (57.5%), while only 12.5% of them chose chocolate pellets. The remaining 30% of the animals, the so-called *undecided*, failed to choose between the presented rewards within the 5-testing min. Interestingly, 72.5% of VEH and 42.5% of CORT-treated animals chose the grain-based reward. The individuals that chose the unfamiliar, chocolate reward represented 22.5% of the control animals as opposed to only 2.5% of the CORT-treated mice. Noteworthy, 55% of CORT-treated animals did not select an option, while only 5% of VEH-treated animals failed to choose between options before the end of the task. The distribution of CORT- and VEH-treated mice between the three possible classes was compared, highlighting a significant difference (*X*^2^, *p* = 0.0000), which is mainly accounted for by the chocolate reward and undecided choices (see Figure 4A).

Along the same line, the latency of the first choice was compared between the different conditions, showing that VEH-treated (*n* = 38) and CORT-treated (*n* = 18) animals, if they chose, required a similar amount of time to select their first pellet [latency of the first choice (s) ± SEM, VEH: 62.90 ± 12.17; CORT: 71.93 ± 20.22] (*t*_54_ = −0.4, *p* = 0.69; GBW, *X*^2^ = 0.5, *p* = 0.50; Figures 4B,C). When considering reward types separately, no differences were found concerning the latency to be chosen [mean choice latency (s) ± SEM, grain-based: 57.96 ± 10.87 chocolate: 101.87 ± 28.67] (*t*_54_ = 1.6, *p* = 0.11), neither an effect of the pharmacological treatment [grain based, VEH: 54.73 ± 13.16; CORT: 63.47 ± 19.48; chocolate, void] (*t*_44_ = −0.4, *p* = 0.70). Additionally, the total number of eaten pellets was compared between conditions, evidencing a significantly reduced intake in the CORT condition [reward intake ± SEM, VEH: 9.48 ± 0.81; CORT: 3.33 ± 0.68] (*t*_78_ = 5.8, *p* = 0.0000; Figure 4D). Combined, these results are consistent with those obtained for the NSF (longer latencies to start eating) and PR (reduced effort allocation) tasks, showing altered appetitive and consummatory behaviour after chronic exposure to CORT. They, therefore, point to disrupted reward appreciation with CORT-induced exacerbation of the natural food neophobia of rodents.

The same mice (VEH, *n* = 40; CORT, *n* = 40) were re-tested in the FFT paradigm a week after the first behavioural assessment. When facing the choice between familiar rewards, the majority of individuals continued to choose primarily the grain-based option (57.5%). However, the choice of chocolate pellets increased to 37.5%. Noteworthy, *undecided* represented only 5% of the animals. Individuals opting for the non-chocolate reward represented 62.5% of VEH and 52.5% of CORT-treated animals. The chocolate reward was chosen by 37.5% of VEH and CORT-treated mice. Notably, the *undecided* group was only comprised by CORT-treated animals. As previously, the distribution of the individuals of the CORT and VEH conditions into the three possible classes was compared. In this re-testing phase, CORT-treated animals chose the chocolate reward as frequently as control animals, but the distribution of the *undecided* group was significantly different compared with the results of the neophobic testing (*X*^2^, *p* < 0.05; Figure 4E).

Regarding the latency to choose, the individuals from both conditions required less time to select their first reward compared with the first testing session (FFT-neophobia vs FFT-re-test, VEH: *t*_37_ = 4.5, *p* < 0.0001; CORT: *t*_17_ = 3.2, *p* < 0.01; GBW, VEH: *X*^2^ = 47.8, *p* < 0.0001; CORT: *X*^2^ = 16.3, *p* < 0.0001; Figures 4F,G). When compared, CORT-treated (*n* = 36) and VEH-treated (*n* = 40) animals did not significantly differ concerning the time they required to select their first reward [latency of the first choice (s) ± SEM, VEH: 9.64 ± 1.14; CORT: 25.02 ± 8.89] (*t*_74_ = −1.8, *p* = 0.07; GBW, *X*^2^ = 1.3, *p* = 0.25; Figures 4H,I). However, when each reward type was considered separately, the choice latency for the chocolate option [mean choice latency (s) ± SEM: 30.22 ± 10.50] was found to be significantly longer than the one for the grain-based option [8.26 ± 0.82] (*t*_74_ = 2.6, *p* < 0.05). This difference was not found attributable to the pharmacological treatment (VEH vs CORT, grain-based: *Z* = −0.6, *p* = 0.56; chocolate: *Z* = −1, *p* = 0.31). Furthermore, the total food intake over the task continued being lower in CORT-treated animals [reward intake ± SEM, VEH: 14.58 ± 0.74; CORT: 11.70 ± 1.01] (*t*_78_ = 2.3, *p* < 0.05; Figure 4J).

Hedonic appreciation in mice was then evaluated through their consummatory behaviour in the SPT. The corresponding results demonstrate that all individuals expressed a strong preference for the sucrose solutions compared to water, independent of the sucrose concentration (consumption of sucrose solution vs 50%: all *ps* < 0.0001). No differences were found between CORT-treated mice and the individuals from the control group concerning their consummatory behaviour, independently of the concentration of the sucrose solution (2.5% sucrose solution: *t*_78_ = 1.8, *p* = 0.08; 0.8% sucrose solution, UMW: *Z* = 0.5, *p* = 0.60; see Figure 4K). Hence, the SPT did not evidence altered reward processing or hedonic underappreciation in the mice after chronic CORT exposure.

Combined, these results do not evidence a significant impairment of reward valuation in mice following chronic CORT administration when food rewards are familiar, but they do indicate an obstruction of behavioural initiation reminiscent to human abulia. Moreover, the reduction of reward intake in CORT-treated animals during the FFT shows an impact of the GC pharmacological treatment on consummatory behaviour, although not confirmed by the SPT evaluation. In order to better understand this difference, the total consumption of sucrose solution (2.5%) and the total intake during the FFT re-test phase were directly compared. A significant correlation between SPT and FFT scores was obtained (*r* = 0.4274, *p* < 0.0001), indicating that chronic CORT administration might affect consummatory behaviour in mice, suggesting that SPT assessment at 2.5% might not be adapted to reveal this effect.

**FIGURE 5:**
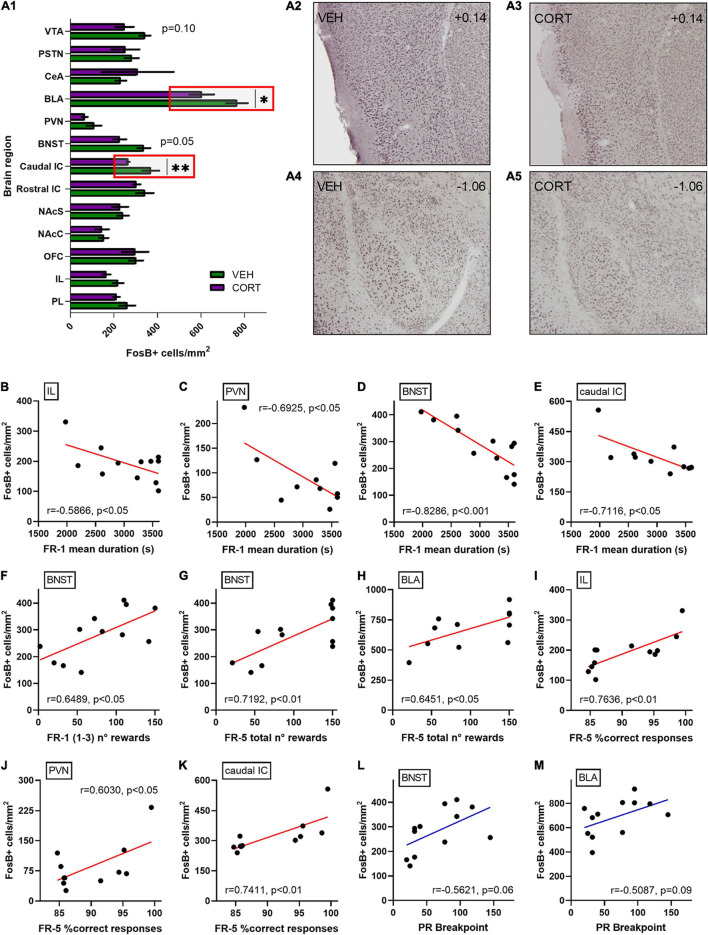
Relative quantification of FosB-labelled cells densities in various discrete brain regions of interest following effort-allocation testing in a PR schedule of reinforcement task. **(A1)** FosB quantification is expressed as mean of number of immunoreactive-labelled cells per surface (mm^2^). Similar activation patterns were found in the control and CORT-treated animals in the following brain regions: the PL (VEH: 261.31 ± 36.82; CORT: 211.77 ± 15.14) and IL cortices (VEH: 218.61 ± 25.19; CORT: 165.12 ± 18.75); the OFC (VEH: 301.67 ± 32.81; CORT: 297.65 ± 61.01); the NAcC (VEH: 153.46 ± 21.15; CORT: 145.10 ± 31.73) and NAcS (VEH: 241.737 ± 26.34; CORT: 228.17 ± 36.96); the rostral aIC (VEH: 342.54 ± 39.72; CORT: 304.36 ± 17.50); the PVN (VEH: 108.68 ± 33.85; CORT: 66.02 ± 13.20); the CeA (VEH: 231.37 ± 25.40; CORT: 308.48 ± 165.63); and the PSTN (VEH: 281.70 ± 32.64; CORT: 252.22 ± 64.90). However, a significant effect of the GC pharmacological treatment was evidenced in **(A2,3)** the caudal aIC (**p < 0.01) and the BLA (*p < 0.05) **(A4,5)**, with reduced activation in the CORT-treated animals. Simultaneously, two negative trends of activation were found in the CORT animals in the BNST (*p* = 0.05) and the VTA (*p* = 0.10). Among overall data (VEH + CORT individuals), significant negative correlations were found between FR-1 mean duration of the sessions and FosB-labelled cells densities in **(B)** the IL cortex (*p* < 0.05), **(C)** the PVN (*p* < 0.05), **(D)** the BNST (*p* < 0.001), and **(E)** the caudal aIC (*p* < 0.05). **(F)** The number of rewards earned in the three first FR-1 sessions also significantly correlate with neurobiological scores in the BNST (*p* < 0.05). Concerning the FR-5 phase, the number of earned rewards significantly correlate with the neurobiological scores **(G)** in the BNST (*p* < 0.01) and **(H)** the BLA (*p* < 0.05), as the mean percentage of correct responses does **(I)** in the IL (*p* < 0.01), **(J)** the PVN (*p* < 0.05), and **(K)** the caudal aIC (*p* < 0.01). No significant correlation was found between breakpoint (BP) scores in the PR schedule of reinforcement task and FosB-labelled cells densities, irrespective of the brain region considered, but two trends **(L)** in the BNST (*p* = 0.06) and **(M)** in the BLA (*p* = 0.09), pointing to an intricate relationship between neural activation patterns and effort allocation valuation as a major motivational component in the control and CORT-treated animals.

Behavioural results were then related to neural patterns of activation in the brain regions of interest, aiming at identifying neural correlates for the CORT-induced behavioural modifications. The levels of chronic neural activation addressed by FosB expression after the PR testing are presented in Figure 5A. The density of FosB-labelled cells was significantly decreased in the caudal aIC and the BLA of CORT-treated animals [mean of FosB + cells per mm^2^ ± SEM, caudal aIC: 265.31 ± 6.46; BLA: 603.71 ± 56.07] compared with the control individuals [caudal aIC: 369.14 ± 38.85; BLA: 765.78 ± 49.34] (UMW, caudal aIC: *Z* = 2.7, *p* < 0.01; BLA: *Z* = 2.1, *p* < 0.05). A negative trend of reduced FosB-labelled cells density was also evidenced in the dorsal BNST [VEH: 337.41 ± 29.97; CORT: 227.11 ± 29.81] (*Z* = 1.9, *p* = 0.05) and the VTA [VEH: 342.43 ± 26.04; CORT: 249.05 ± 41.85] (*Z* = 1.6, *p* = 0.10) of CORT-treated animals. Otherwise, no differences between conditions were found in the other brain regions here studied (*Z* < 1.6, all *ps* > 0.20). Nevertheless, the vast SEM values obtained for the CeA of the CORT condition suggest that this dataset should be replicated or focused on anatomical subdivisions (Barbier et al., 2020) in order to circumvent inaccurate interpretation. Therefore, these results suggest that the GC pharmacological treatment might have impacted effort and reward valuation and processing during PR testing by inducing aberrant functional neural activation and plasticity on aIC and BLA, respectively.

Since a detrimental effect of the GC pharmacological treatment was evidenced on instrumental acquisition in the FR-1 schedule of reinforcement, FosB-labelled cells densities obtained for the different brain regions of interest were compared to FR-1 behavioural scores aiming to substantiate this finding (see Figures 5B–K). No significant direct correlations were found between the number of FR-1 sessions and neurobiological scores, irrespective of the brain region investigated (*r*, all *ps* > 0.10). However, the mean duration of FR-1 sessions negatively correlates with FosB-labelled cells densities in the IL cortex (*r* = 0.5866, *p* < 0.05), the PVN (*r* = −0.6925, *p* < 0.05), the BNST (*r* = −0.8286, *p* < 0.001) and the caudal aIC (*r* = −0.7116, *p* < 0.05), and tends to negatively correlate in the BLA (*r* = −0.5454, *p* = 0.07). Moreover, the rewards earned in the first three FR-1 sessions significantly correlate with neurobiological scores in the BNST (*r* = 0.6489, *p* < 0.05) and tend to correlate in the PVN (*r* = 0.5254, *p* = 0.10). Similarly, the rewards earned during the FR-5 phase correlate with the neurobiological scores in the BNST (*r* = 0.7192, *p* < 0.01) and the BLA (*r* = 0.6451, *p* < 0.05), and tend to correlate in the caudal aIC (*r* = 0.5888, *p* = 0.06) and the VTA (*r* = 0.5556, *p* = 0.08). Finally, mean percentages of correct responses during the FR-5 significantly correlate with FosB-labelled cells densities in the IL cortex (*r* = 0.7636, *p* < 0.01), the PVN (*r* = 0.6030, *p* < 0.05) and the caudal aIC (*r* = 0.7411, *p* < 0.01), and tend to correlate in the BNST (*r* = 0.5007, *p* = 0.10). These results suggest the involvement of multiple discrete brain regions in food-related associative learning deficits, potentially modulating subsequent motivation for effort allocation in mice.

Breakpoint scores were then systematically compared to FosB-labelled cells densities, aiming at directly appraising the neuronal bases of differential animal motivation in the PR paradigm. Despite the fact that no significant correlations were found, including analyses of the caudal aIC (*r* = 0.4353, *p* = 0.18) and the VTA (*r* = 0.5094, *p* = 0.11), two positive trends were evidenced for BP scores and FosB-labelled cells densities in the BNST (*r* = 0.5621, *p* = 0.06; Figure 5L) and the BLA (*r* = 0.5087, *p* = 0.09; Figure 5M). These results, therefore, suggest a more intricate relationship between neural activation patterns and effort allocation in the PR task specifically, and point toward the influence of the BLA as a modulator of mice effort-based motivation in physiological conditions and upon chronic distress.

## Discussion

The results of this experimental investigation show that chronic GC exposure has complex detrimental influences on motivational components subserving goal-directed behaviours and specifically, on appetitive and consummatory aspects involved in effort and reward valuation. These motivational deficits likely ground on alterations of interoceptive and sensory cue-related information processing, from neural network computations in somatosensory and associative cortices with neuromodulatory influences of the so-called “brain reward system”.

The present investigation studied various complementary motivational components through food rewarding tasks, allowing to address hedonic valuation and appreciation of the reinforcers. Altered appetitive behaviour consistent with the “wanting” category mechanism of incentive motivation (Berridge and Kringelbach, 2015) was observed in the CORT-treated animals, as shown by the increased latency to feed in the NSF task, the decreased effort allocation in the PR task and the increased frequency of *undecided* individuals as assessed in the FFT. These results, therefore, demonstrate an effect of high-circulating GC plasma levels on the ability of animals to initiate behavioural actions in a similar manner to human abulia (Husain and Roiser, 2018) and suggest an impairment of incentive salience neural mechanisms (Berridge, 2018). Besides, this could also reflect psychomotor deficits reminiscent to those observed in neuropsychiatric conditions in accord with our previous preclinical (Cabeza et al., 2021) and clinical data (Bennabi et al., 2013). Thus, this result adds value to data suggesting that chronic CORT exposure yields mice especially vulnerable to action-outcome encoding and further consolidation in food-reward-based tasks, in accord with our previous results on value-based decision-making under uncertainty (Cabeza et al., 2021).

The study of positive valence-based motivated behaviours in rodents is valuable for translational research on reinforcement learning, but unfortunately, literature is incongruent with regard to findings concerning the CORT model. As chronic CORT administration induces a consistent negative valence phenotype (David et al., 2009; Dieterich et al., 2019), not all aspects of hedonic appreciation, including reward valuation, are reportedly altered (Cabeza et al., 2021). Grounded on previous studies showing different consummatory scores in healthy mice displaying different gambling strategies (Pittaras et al., 2016), this study sought for new scientific evidence of distress-induced underappreciation of reward as a modulator of decisional strategies. Chronic GC administration did not differentially impact reward choice of animals in the FFT, suggesting similar hedonic valuation, but the overall food intake was lower in the CORT-treated animals. Besides, SPT scores did not differ across pharmacological conditions. Thus, chronic GC exposure may affect hedonic reward appreciation through restraining initiation of actions rather than through obstruction of reward valuation. This apparent discrepancy in the motivational “liking” category mechanism (Berridge and Kringelbach, 2015) might result from protocol variation, including different test durations, and even though various sucrose concentrations have been used in this study, ceiling effects on palatability cannot be excluded. Further investigations are, therefore, necessary to better disentangle the contribution of hedonic impact on adaptive decisional capability. Moreover, it is possible that food deprivation enhanced incentive valuation differently in the animals across pharmacological conditions; thus, further investigations should address this experimental limitation.

Interestingly, the FFT paradigm, which allows evaluating hedonic appreciation and particularly reward valuation, successfully suited the study of additional behavioural aspects potentially influencing goal-directed behaviours. We demonstrated that chronic GC exposure exacerbates food neophobia in mice, and most importantly, it prompts indecision. Animals treated with CORT more frequently vacillated or had difficulty in initiating their choice between food rewards when one option was unfamiliar to them.

The operant-based experimental design used in the present investigation enabled revealing a delayed formation of action-outcome associations in individuals chronically exposed to high-circulating levels of GC, in accord with an altered exploration-exploitation trade-off demonstrated in a valued-based decision-making task (Cabeza et al., 2021). The CORT-induced dysfunctional amotivated state in animals, as shown by the decrease in effort allocation for reward and which resembles to human apathy, further substantiates these findings.

Through impacting action and outcome valuation, chronic GC exposure might obstruct instrumental association encoding and subsequently alter behavioural adaptability in dynamic environments, yielding to poor operational learning. It is, therefore, plausible that CORT-induced altered reward/effort valuation, processing, and integration will impact reward responsiveness through reinforcement learning in valued-based decisional tasks. Chronic GC exposure would, therefore, reduce the learning capabilities of mice, potentially hampering the integration of environmental contingencies necessary to long-term beneficial performance. However, future investigations should measure other informative parameters, such as perseverative actions in order to better understand how chronic CORT impacts learning.

The discrete “localisationist” approach here used to study the neurobiological underpinnings of CORT-induced motivational deficits revealed a pharmacological impact on neural activation of selected discrete brain regions involved in effort and reward processing (Gogolla, 2017; Beyeler et al., 2018). Anterior IC and BLA neural activations were decreased subsequent to long-lasting exposure to GC. This study used different coordinates within the rodent aIC in order to address reward-related processing and the representation of anticipatory cues (Kusumoto-Yoshida et al., 2015; Gehrlach et al., 2020) as essential processes of decision-making. Indeed, the IC participates in decision-making under uncertainty (Clark, 2010), and the aIC, in particular, is involved in contingency acquisition on rodent gambling tasks by modulating reward preference for punishment (Pushparaj et al., 2015). The IC is also relevant to its contribution to initiating feeding behaviour as a part of a basal ganglia-like network (Barbier et al., 2020), depending on cognitive and emotional influences. Hence, chronic GC exposure could impact the motivational state of animals by interfering with aIC-related neural network activity, and disrupting the instrumental behaviour associated with food delivery in the PR paradigm. The CORT-induced aberrant aIC neuronal activity, here identified, might reflect altered anticipatory functioning that curtails reward expectation processing and consequent conditioned responses. However, the lack of significant correlation between BP scores and discrete caudal aIC FosB-labelled cells densities pleads for more complex than direct causality relationships.

There is also strong evidence for the prominent role of the BLA in appetitive learning and conditioning by encoding motivational information related to instrumental association, and for its contribution during effort-based rewarding tasks (Wassum and Izquierdo, 2015; Beyeler et al., 2018; Ferland et al., 2018). In this regard, chronic GC exposure might impact BLA neural activity, obstructing the motivation of animals to effort allocation for food reward likely by disrupting reward-value encoding. Moreover, the positive trend found in the correlation between BP scores and BLA FosB-labelled cells densities aligns with this hypothesis and could also explain the trend found for the BNST. This considers the involvement of the unidirectional excitatory BLA-to-BNST projections in behavioural output signalling. The BNST, in addition to its contribution to behavioural signalling, is highly responsive to stress and has been associated with distress-related behavioural dysfunction *via crf* signalling (Gray et al., 1993; Ulrich-Lai and Herman, 2009; Hu et al., 2020). However, further investigation is necessary in order to characterise the neural cell types involved in the activity changes identified. Further clustering analyses based on the neurobiological data will also be useful for identifying neurobiological biomarkers predictive of particular behaviours. Combined, these results further substantiate that CORT-treated animals show a compromised motivational state rooted in aberrant aIC and BLA-related neural networks activations involved in effort and reward processing, which could hamper decision-making processing.

Unexpectedly, the neural activation pattern in the NAc was not significantly impacted by sustained exposure to GC, even if a recent study suggests its causal role in instrumental reward seeking in the CORT model (Dieterich et al., 2021). Similarly, a stronger effect of the GC pharmacological treatment was expected on the VTA neural activation, since its contribution is considered crucial to the modulation of reward-motivated behaviours *via* associative learning (Bouarab et al., 2019). Ongoing analysis aiming at addressing these points of conflict should help clarify the contribution of the discrete regions of interest and the networks they are part of, to behavioural adaptability in physiological and distress-related conditions.

Modelling distress conditions in animals results, for instance, in disruption of fronto-striatal cognition (Uribe-Mariño et al., 2016; Hupalo et al., 2019) and compromises positive valence systems (Birnie et al., 2020) through alteration of *crf* signalling. In a previous study, we have demonstrated that chronic CORT exposure induces suboptimal decision-making in male mice (Cabeza et al., 2021), and we have suggested that this alteration relates to high mPFC CRF levels. Here, we demonstrated that chronic CORT also prompts reward and effort processing and learning deficits in male mice. The results of this investigation are, therefore, in accord with clinical data on motivational deficits (Darcet et al., 2014; Salamone et al., 2018) and suggest that appetitive aspects of motivated behaviours are key players in the modulation of decision-making, particularly in dynamic environments. However, future studies should examine the chronic CORT-induced effects on motivation and decision-making in female mice, since the GC pharmacological treatment differentially induces the emergence of negative valence behaviours across sexes (Mekiri et al., 2017; Yohn et al., 2019).

In sum, the presented results provide novel insight into the mechanisms of motivational-based learning impairments induced by chronic exposure to GC, and support the validity of the chronic CORT model for translational investigation on effort and reward processing across species and multiple discrete clinical conditions associated with chronic distress. The results are, therefore, relevant to understanding the pathophysiological mechanisms underlying amotivated-based behaviours in a transdiagnostic perspective.

## Data Availability Statement

The raw data supporting the conclusion of this article will be made available by the authors, without undue reservation.

## Ethics Statement

The animal study was reviewed and approved by Ethical Committee in Animal Experimentation from Besançon (CEBEA-58; A-25-056-2).

## Author Contributions

LC, YP, and DF conceived the experimental design and agreed to be accountable for all aspects of the work in ensuring that questions related to the accuracy or integrity of any part of the work are appropriately investigated and resolved. LC, BR, SC, and CH contributed to the data acquisition. LC and YP analysed the data and wrote the manuscript. All authors critically revised the work and approved the version to be published.

## Conflict of Interest

The authors declare that the research was conducted in the absence of any commercial or financial relationships that could be construed as a potential conflict of interest.

## Publisher’s Note

All claims expressed in this article are solely those of the authors and do not necessarily represent those of their affiliated organizations, or those of the publisher, the editors and the reviewers. Any product that may be evaluated in this article, or claim that may be made by its manufacturer, is not guaranteed or endorsed by the publisher.
